# Automated evaluation of typical patient–ventilator asynchronies based on lung hysteretic responses

**DOI:** 10.1186/s12938-023-01165-0

**Published:** 2023-10-24

**Authors:** Yuhong Chen, Kun Zhang, Cong Zhou, J. Geoffrey Chase, Zhenjie Hu

**Affiliations:** 1 Intensive Care Unit, The Fourth Hospital of Hebei Medical University, Shijiazhuang, China; 2 Department of Mechanical Engineering & Centre for Bio-Engineering, University of Canterbury, Christchurch, New Zealand; 3 Taicang Yangtze River Delta Research Institute, Suzhou, China

**Keywords:** Patient–ventilator asynchrony, Mechanical ventilation, PV loop, Hysteretic lung mechanics, Hysteresis loop analysis, Intensive care unit

## Abstract

**Background:**

Patient–ventilator asynchrony is common during mechanical ventilation (MV) in intensive care unit (ICU), leading to worse MV care outcome. Identification of asynchrony is critical for optimizing MV settings to reduce or eliminate asynchrony, whilst current clinical visual inspection of all typical types of asynchronous breaths is difficult and inefficient. Patient asynchronies create a unique pattern of distortions in hysteresis respiratory behaviours presented in pressure–volume (PV) loop.

**Methods:**

Identification method based on hysteretic lung mechanics and hysteresis loop analysis is proposed to delineate the resulted changes of lung mechanics in PV loop during asynchronous breathing, offering detection of both its incidence and 7 major types. Performance is tested against clinical patient data with comparison to visual inspection conducted by clinical doctors.

**Results:**

The identification sensitivity and specificity of 11 patients with 500 breaths for each patient are above 89.5% and 96.8% for all 7 types, respectively. The average sensitivity and specificity across all cases are 94.6% and 99.3%, indicating a very good accuracy. The comparison of statistical analysis between identification and human inspection yields the essential same clinical judgement on patient asynchrony status for each patient, potentially leading to the same clinical decision for setting adjustment.

**Conclusions:**

The overall results validate the accuracy and robustness of the identification method for a bedside monitoring, as well as its ability to provide a quantified metric for clinical decision of ventilator setting. Hence, the method shows its potential to assist a more consistent and objective assessment of asynchrony without undermining the efficacy of the current clinical practice.

## Background

Mechanical ventilation (MV) is the standard therapy for intensive care unit (ICU) patients with respiratory failure [32], and was one major therapy during the Covid-19 pandemic [28, 30, 54]. The major goal of MV is to support patient breathing to allow recovery and improve outcomes. However, optimizing patient-specific ventilator setting or modes is difficult, with few accepted or standardized endpoints or guidelines [32, 36]. Significant inter- and intra-patient variability in lung mechanics and response to MV [8–10, 29] can thus lead to sub-optimal care and outcomes.

In particular, suboptimal MV settings can lead to a mismatch between patient demand and ventilator delivery, defined as patient–ventilator asynchrony. More specifically, asynchrony is caused by poor patient–ventilator interaction when the ventilator supplies of flow, pressure is uncoordinated with patient demands regarding time, magnitude or effort [4, 24, 35]. Patient asynchrony can reduce outcomes, increasing length of ventilation, length of stay, risk of death, and thus cost. The ability to accurately identify and monitor asynchrony incidence and type, in real-time, would provide significant clinical information to both guide and personalise care, ameliorating these issues.

Visual inspection of ventilator waveforms (pressure and/or flow) has been a major approach for bedside asynchrony identification [21, 38], but requires skilled training and experience, and is subjective. However, subjective bedside waveform analysis lacks accuracy and robustness, and is not continuous. In particular, research indicates less than 25% of ICU health professionals could identify all typical types of patient asynchronies [14, 41], where each asynchrony type merits a specific therapeutic strategy to reduce asynchrony and prolonged weaning [24]. Therefore, automated, computer algorithm-based recognition of asynchrony types has been evaluated to overcome subjective bias and the lack of a continuous measurement, and thus improve care and outcomes [7, 22, 37, 39, 40, 55].

Whilst patient asynchrony may occur in various forms, seven types of patient–ventilator interactions have been recognized as the most common asynchronies, in specific, flow asynchrony, ineffective effort, reverse triggering, auto triggering, double triggering, premature cycling, and delayed cycling [14, 20, 21, 24, 38]. However, automated algorithms in the literature focused on identification of only one or a few types of asynchronies, and thus did not generalize well over a wide range of asynchronous scenarios [7, 11, 22, 37, 39, 40, 55].

Patient asynchrony does generate clear, discriminating information in the waveforms [12]. Thus, identification algorithm performance is highly dependent on accurately modelling and capturing these unique waveform characteristics. In searching for effective detection methods, it is important to account for the coupling effect of waveforms during asynchrony identification as the analysis of one-dimension waveform (pressure or flow) may not present the complete information necessary for accurate recognition of all asynchrony types [11, 26, 31]. Even methods analyzing both pressure and flow signals, whilst failing to consider their coupling effect, could still miss some path-dependent mechanics for a unique identification [6, 34, 38].

Hysteresis loops, representing coupled force and deformation relationships, have proven effective for accurately identifying the fundamental mechanics of nonlinear dynamic systems [57, 58]. Importantly, the pressure–volume (PV) loop is equivalent to the nonlinear force and deformation structural hysteresis loop [49, 60]. Particularly, PV loops shows a similar explicit hysteretic mechanism to hysteresis loop, including both pressure, volume data and their variations over breathing history. Therefore, the piecewise regression model for a hysteresis loop can be further used to capture the coupled mechanics observed in PV loop, with a global minimum solution required for an identifiable model, showing its advantage over black-box methods given limited data are available [53].

In addition, traditional model-based methods aim to define mathematical equations and find model parameters governing physical or physiology properties for a fixed system. They are thus more suitable for a relatively fixed pattern of responses, rather than highly nonlinear and highly variable systems. In contrast, model-free methods are more likely necessary for modelling highly nonlinear responses with many potential underlying mathematical functions, but are limited by their lack of explicit physical/physiology meanings with little interpretable knowledge about the internal structure of the defined arbitrary model functions. The hysteresis loop analysis (HLA) method used in this study combines the advantages of traditional model-based and model-free methods to explicitly examine a range of nonlinear responses without requiring a single fixed mathematical equation, further enabling an automated assessment of asynchrony directly via ventilator-collected pressure and flow data.

Research based on PV loop has shown its promising utilization for modelling, identification and prediction of fully ventilated breath cycles [47, 48, 50, 59]. More specifically, Zhou et al. developed a nonlinear hysteretic lung mechanics model (HLM) combined to HLA to replicate the fully ventilated respiratory response without considering asynchrony [59]. The HLM model was further combined with identification method to reconstruct complete ventilated breathing cycles removing the impact of asynchronous patient effort [61], showing the potential to identify various forms of asynchronies via PV loop analysis. However, the explicit relationship of unique patterns of PV loop over common asynchrony types remains to be interpreted for all types of asynchronies, which would be of significant clinical utility.

Therefore, this study proposed a hysteresis loop analysis method based on piecewise regression linear model to explicitly represent the patterns of seven types of asynchronies. The goal of developing this method is to automate the inspection and analysis using measured PV loops constructed from the recorded pressure and flow/volume waveforms with competitive efficacy to visual inspections. The specific patterns of 7 most common major asynchrony types were defined via PV loops with piecewise regression models. Finally, the incidence and types of asynchronies were identified for clinical data of 11 patients with comparison to human inspection as a ground truth, with results presented as sensitivity and specificity.

## Results

Table 1 shows per-patient and overall performance in detecting asynchrony. In Table 1, the count of asynchrony includes cases of TP when identified as asynchrony for asynchrony breath, and FN when identified asynchrony for non-asynchrony breath. In addition, the count of non-asynchrony includes cases of TN when identified as non-asynchrony for non-asynchrony breath, and FP when identified non-asynchrony for asynchrony breath. Sensitivity and specificity are then calculated using Eqs. (12) and (13), respectively. The accuracy is calculated using Eq. (14).

**Table 1:** Identification performance compared to clinical inspection for each patient and overall without subdividing by asynchrony type, which is in Table 3

Patient	Total breaths	# Asynchronous	# No asynchrony	Sensitivity	Specificity	Accuracy
1	500	154	346	99.4%	99.4%	99.4%
2	500	22	478	95.5%	99.8%	99.6%
3	500	89	411	92.1%	99.3%	98.0%
4	500	496	4	99.0%	N/A	98.6%
5	500	274	226	93.8%	99.6%	96.4%
6	500	231	269	94.8%	93.7%	94.2%
7	500	218	282	99.5%	96.1%	97.6%
8	500	433	67	94.9%	92.5%	94.6%
9	500	216	284	97.2%	97.5%	97.4%
10	500	10	490	90.0%	97.1%	97.0%
11	500	0	500	N/A	100.0%	100.0%
Overall	5500	2143	2857	96.6%	97.8%	97.3%

Table 2 shows the performance for each type of asynchrony. Overall identification accuracy is high ranging from 94.2 to 100% per-patient (97.3% over all patients), and, equally so for asynchrony type, ranging from 97.4 to 99.6%. Specificity results are slightly higher than accuracy, and sensitivity is slightly lower accordingly. Overall, these results show significant robustness across patients, asynchrony types, and incidence rates per patient. High accuracy implies clinical decisions would not be altered.

**Table 2:** Identification performance compared to clinical inspection by type of asynchrony and for non-asynchrony breaths

Asynchrony types	TP	FN	TN	FP	Sensitivity (%)	Specificity (%)	Accuracy (%)
fa	68	8	4912	12	89.5	99.8	99.6
rt	595	13	4384	8	97.9	99.8	99.6
pc	383	22	4581	14	94.6	99.7	99.3
dt	241	5	4748	6	98.0	99.9	99.8
dc	202	15	4741	42	93.1	99.1	98.9
ie	133	9	4848	10	93.7	99.8	99.6
at	415	34	4543	8	92.4	99.8	99.2
Non-asynchrony breaths	2542	52	2328	78	98.0	96.8	97.4

It is worth noting, the sensitivities for P3, P5 and P8 are relatively lower than other patients due to the low magnitude of asynchronous segment in the PV loop, which were not successfully identified by the algorithm for pc, dc and ie types. The identification of pc, dc and ie types depends on the change of magnitude for their pattern recognition, as seen in Fig. 14, which thus can be misidentified as non-asynchrony given too small change of magnitude. In addition, the lower sensitivity for P10 is because there are 9 out of 10 asynchronous breaths successfully identified, whilst only yielding a sensitivity of 90% because of the small total asynchrony breath number.

**Fig. 1:**
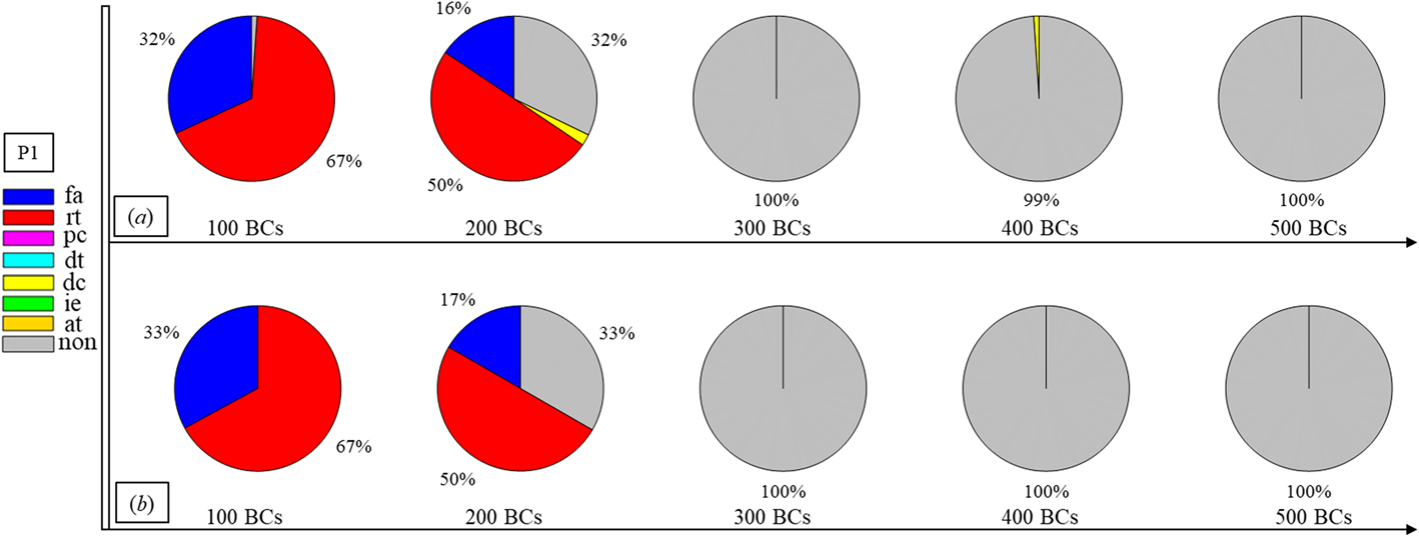
Statistical analysis of asynchrony for P1 over time (every 100 breaths) with comparison between **a** identification algorithm and **b** clinical inspection, where an RM begun at BC282, the 282nd breathing cycle

**Fig. 2:**
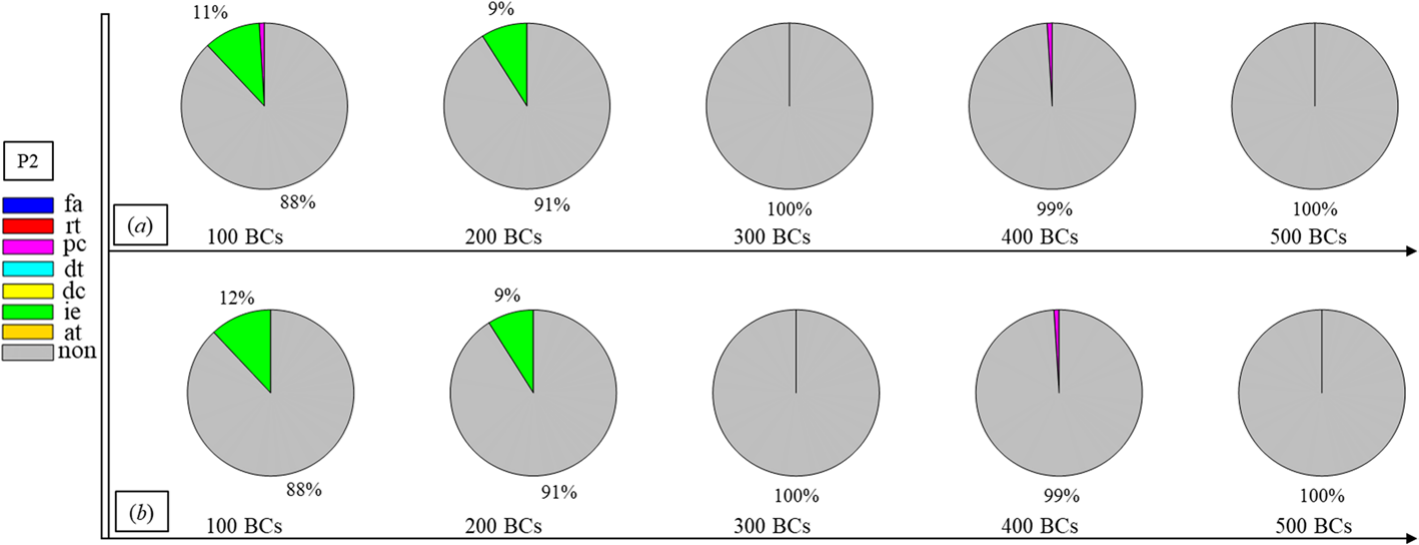
Statistical analysis of asynchrony for P2 over time (every 100 breaths) with comparison between **a** identification algorithm and **b** clinical inspection, where an RM begun at BC325, the 325th breathing cycle

**Fig. 3:**
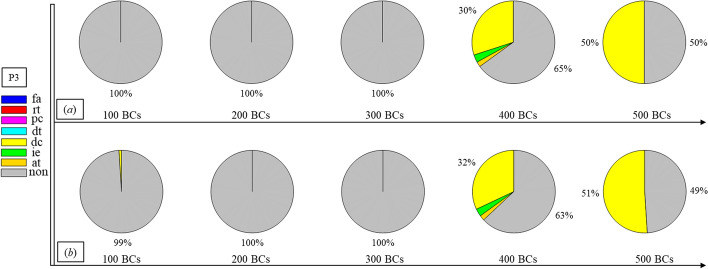
Statistical analysis of asynchrony for P3 over time (every 100 breaths) with comparison between **a** identification and **b** inspection, where an RM begun at BC325, the 325th breathing cycle

**Fig. 4:**
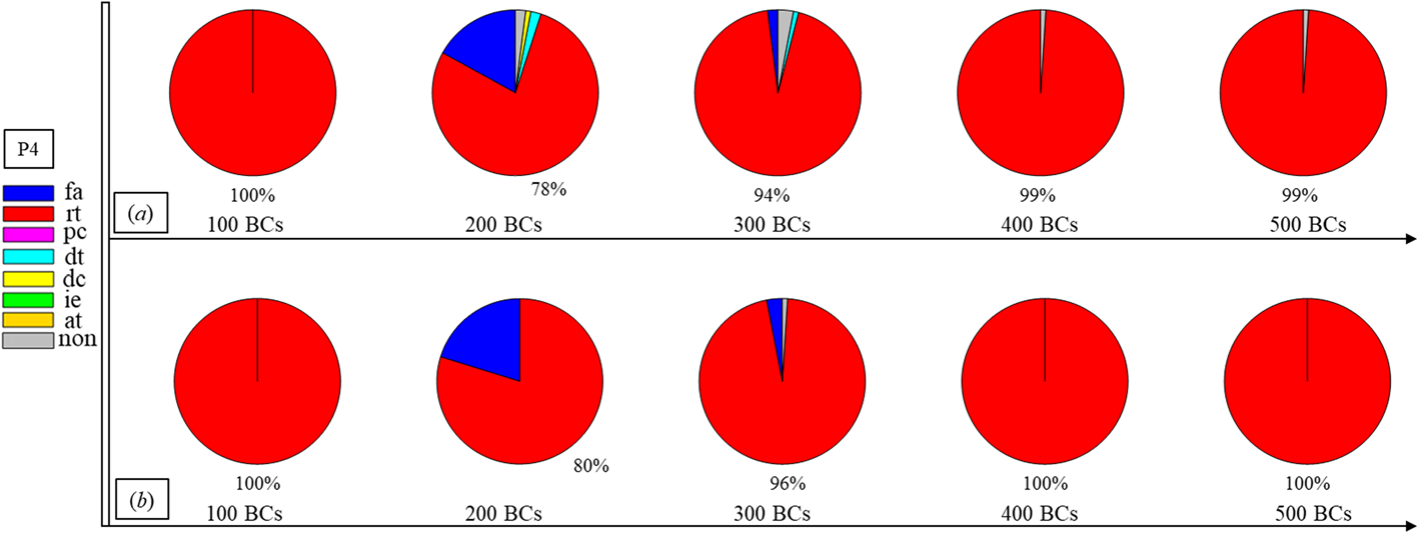
Statistical analysis of asynchrony for P4 over time (every 100 breaths) with comparison between **a** identification algorithm and **b** clinical inspection

**Fig. 5:**
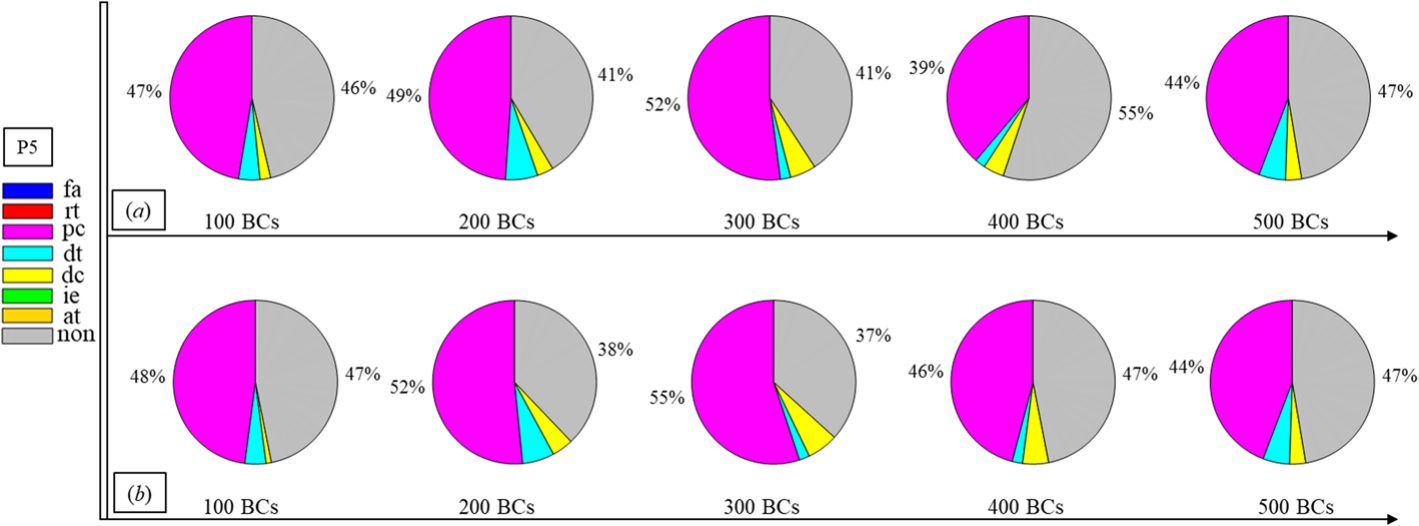
Statistical analysis of asynchrony for P5 over time (every 100 breaths) with comparison between **a** identification algorithm and **b** clinical inspection

**Fig. 6:**
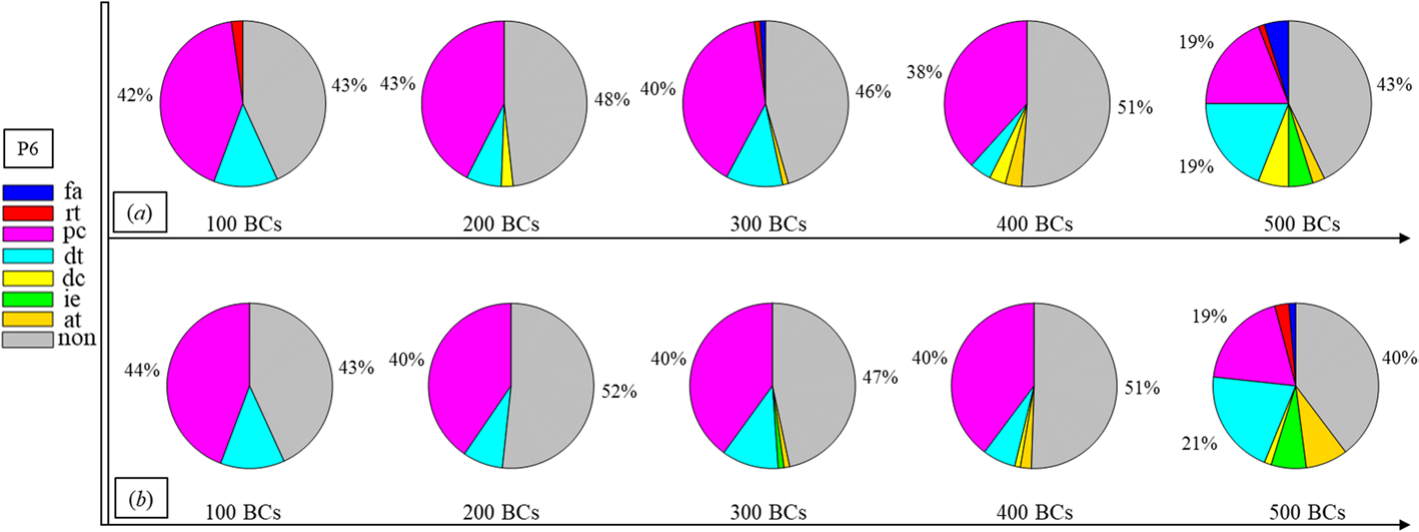
Statistical analysis of asynchrony for P6 over time (every 100 breaths) with comparison between **a** identification algorithm and **b** clinical inspection

**Fig. 7:**
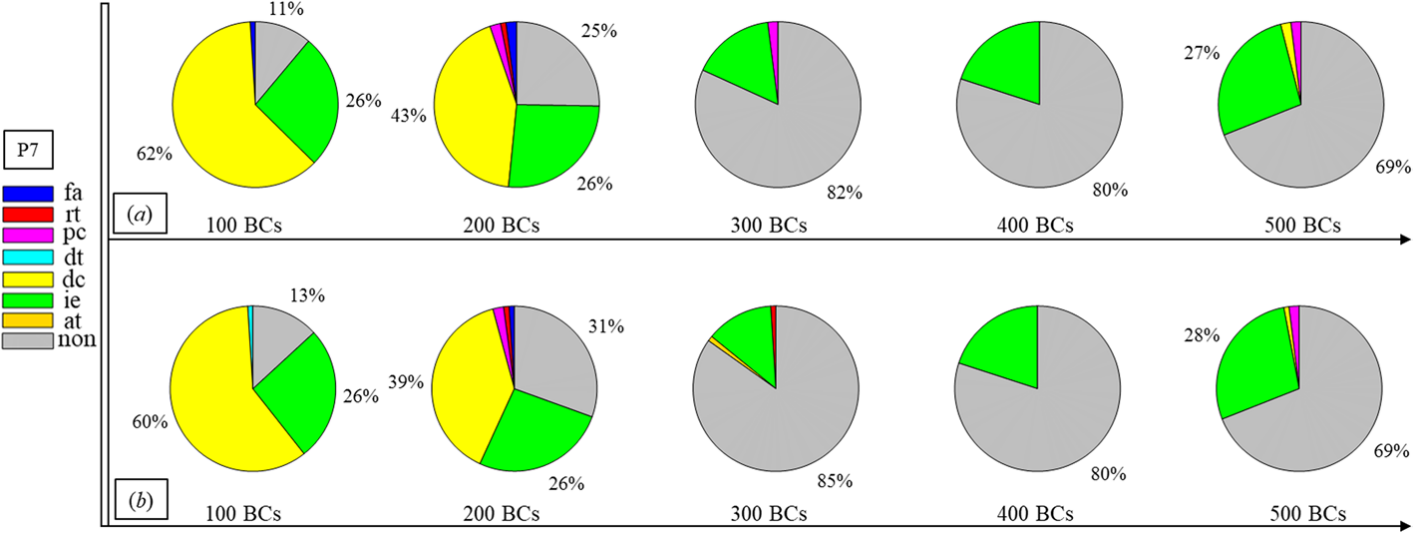
Statistical analysis of asynchrony for P7 over time (every 100 breaths) with comparison between **a** identification algorithm and **b** clinical inspection

**Fig. 8:**
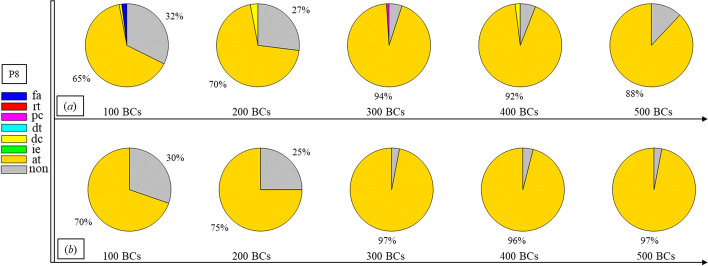
Statistical analysis of asynchrony for P8 over time (every 100 breaths) with comparison between **a** identification algorithm and **b** clinical inspection

**Fig. 9:**
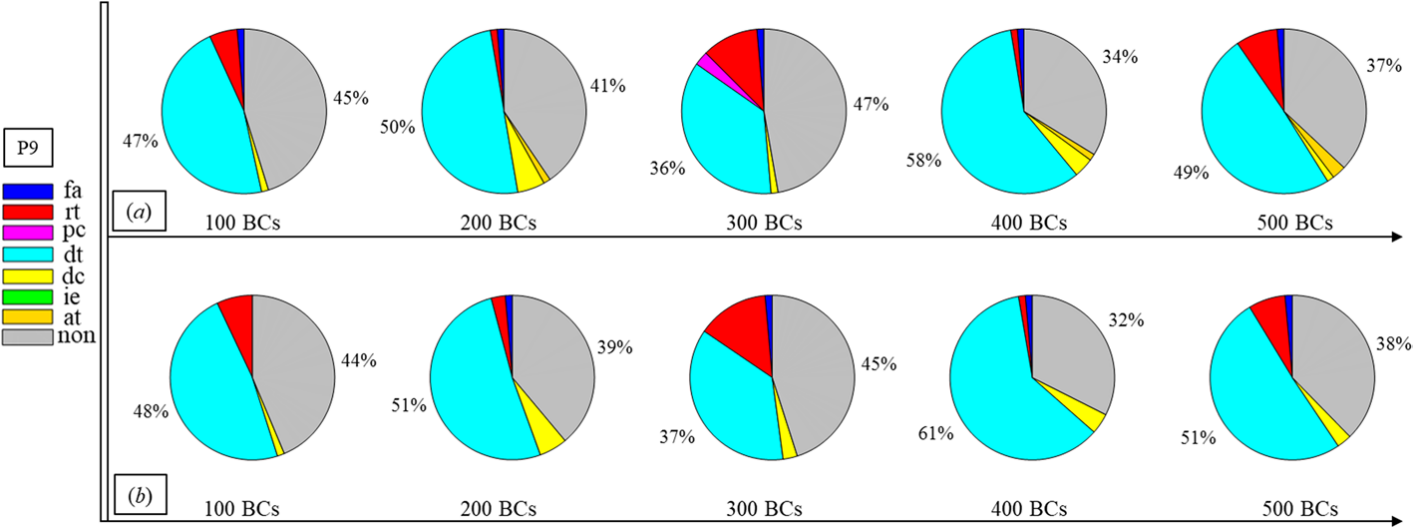
Statistical analysis of asynchrony for P9 over time (every 100 breaths) with comparison between **a** identification algorithm and **b** clinical inspection

**Fig. 10:**
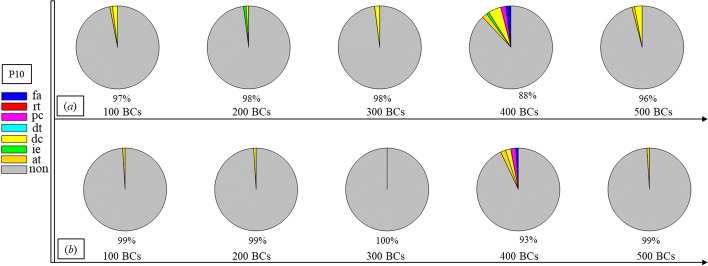
Statistical analysis of asynchrony for P10 over time (every 100 breaths) with comparison between **a** identification algorithm and **b** clinical inspection

**Fig. 11:**
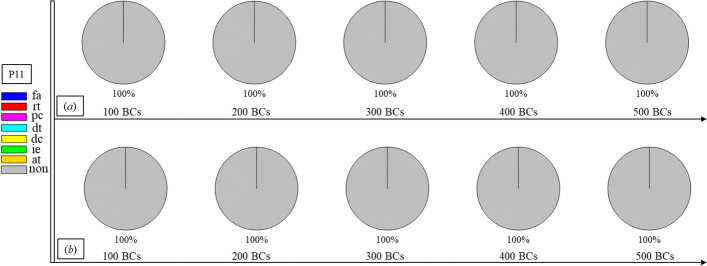
Statistical analysis of asynchrony for P11 over time (every 100 breaths) with comparison between **a** identification algorithm and **b** clinical inspection

Finally, Figs. 1, 2, 3, 4, 5, 6, 7, 8, 9, 10 and 11 present the results for each patient over time (every 100 breaths or ~ 5–7 min), also showing how monitoring would capture trends over time and changes in patient condition over a clinically relevant period [33]. The seven types noted in Figs. 1, 2, 3, 4, 5, 6, 7, 8, 9, 10 and 11 include flow asynchrony (fa), reverse triggering (rt), premature cycling (pc), ineffective effort (ie), double triggering (dt), and delayed cycling (dc), and auto triggering (at).

Note, P1, P2, and P3 underwent recruitment manoeuvre (RM) from breathing cycle (BC) BC282, BC325, and BC513, respectively, as shown in Figs. 1, 2 and 3. In Fig. 1, both identification in Fig. 1a and inspection in Fig. 1b clearly show the intensity of asynchrony decreased from ~ 100% at 100 BCs to ~ 65% at 200 BCs, and finally dropped to ~ 0% at the 300 BCs due to the sedation administration, which is the standard procedure for paralyzing a patient for an RM. This drop-off is thus expected and further validates the model and method’s capability. Similar results for P2 in Fig. 2 reinforce this result. However, sedation appears to cause a worse occurrence of asynchrony for P3 during the RM in Fig. 3. In this case, asynchrony increased near and after the RM. These results match observational trials showing sedation, particularly if it is low, is not necessarily associated with reduced asynchrony and can lead, in some cases, to a higher asynchrony rate [16, 19, 46].

Significant inter- and intra-patient variation is evident for patients P4 to P11, as well, showing the significant variability, which requires automated continuous monitoring. In particular, Fig. 4 for P4 shows consistent reverse triggering and strong and persistent patient effort, over the 5 × 100 breath periods, which may be due to overuse of sedatives, where the increasing sedation is a common first response to reduce asynchrony [18]. Thus, the identification of reverse triggering, separate from other forms of asynchrony, is important to enable an appropriate clinical decision to decrease sedation and change respiratory rate and/or pressure to this specific type of asynchrony. This latter result shows the clinical utility of identifying asynchrony by type in a continuous fashion, as enabled here.

Similarly, for P8 in Fig. 8, significant incidence of auto triggering was identified and observed. This behaviour is caused by airflow obstruction during expiration and is commonly seen in patients with chronic obstructive pulmonary disease or obstructive ventilatory defect [1, 42]. This result matches the clinical diagnosis for P8 in Table 3 with mild obstructive ventilatory defect, and further supports the clinical utility of this model-based monitoring approach.

**Table 3:** Patient demographics

Patient	Sex	Age	Length of MV at data collection	MV mode	Diagnosis
1	Male	62	1 h	PSV	Cardiac cancer surgery
2	Female	70	N/A	VC-AC	ARDS, PF ratio (151)
3	Male	50	N/A	VC-AC	ARDS, PF ratio (194)
4	Male	51	5 h	VC-AC	Right lung space-occupying surgery
5	Male	54	1 day	PC-AC	Intestinal obstruction; digestive tract perforation; septic toxic shock
6	Male	54	1 day	PC-SIMV	Severe pneumonia; acute coronary syndrome
7	Female	74	4 days	VC-AC	Severe pneumonia; acute coronary syndrome
8	Female	72	1 day	PC-SIMV	Stomach cancer; mild obstructive ventilatory defect
9	Male	89	4 days	VC-AC	Digestive tract perforation; Septic shock
10	Male	74	6 days	PC-AC	Septic shock
11	Female	57	44 h	VC-AC	Left pneumonectomy surgery

*PSV* pressure support ventilation, *VC-AC* volume control-assist control, *PC-AC* pressure control-assist control, *PC-SIMV* pressure control-synchronized intermittent mandatory ventilation

## Discussion

Visual inspection of both pressure and flow in real-time, breath-to-breath for human experts is not possible for any length of time. However, PV loops enable automated, simultaneous examination of both pressure and flow via nonlinear hysteresis analysis. Thus, the hysteresis loop analysis was proposed to capture and identify patterns unique to the 7 most common asynchronies, as shown in Fig. 14. Electrical activity of the diaphragm (EAdi) or more advanced methods may provide more accurate asynchrony evaluation. However, visual inspection is still one of the current major ways during bedside monitoring for MV adjustment. Validation against visual inspection of clinical patient data shows high sensitivity and specificity greater than 90% for the proposed method across a range of MV modes, indicating its accuracy and robustness. Monitoring detection over time showed the further, clinically important capability to accurately capture changes in patient state as asynchrony incidence rose or fell over relatively short times, allowing timely clinical recognition, diagnosis, and intervention.

**Fig. 12:**
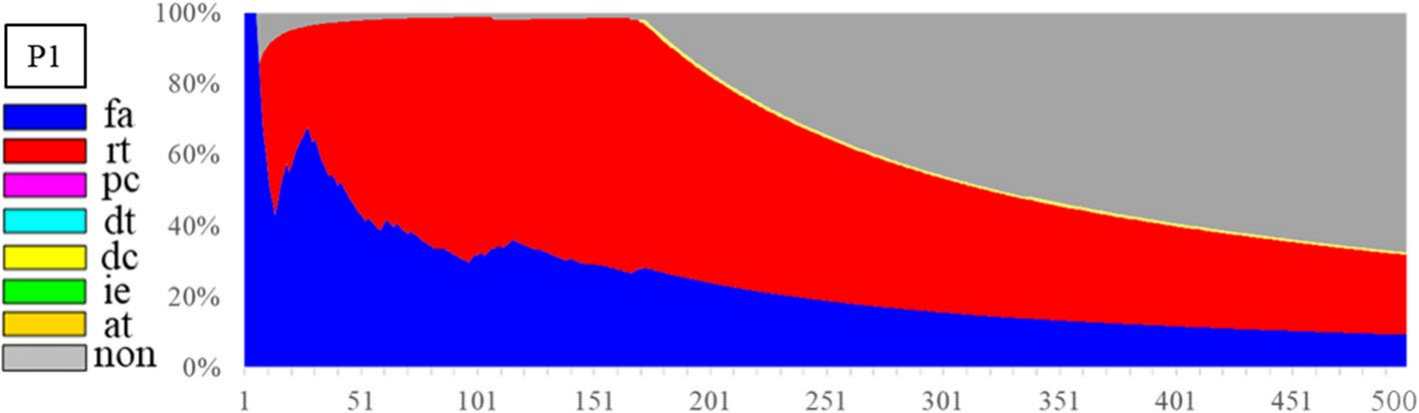
Statistical analysis of asynchrony for P1 over each breath showing declining incidence of asynchrony

In addition, statistical analysis can be conducted per Figs. 1, 2, 3, 4, 5, 6, 7, 8, 9, 10 and 11 providing separated pie distributions for any given period. Equally, Fig. 12 shows a further potential analysis offering consistent assessment over each breath (1–500) during the trial period [5, 52]. There is thus significant potential clinical monitoring and clinical utility enabled by this readily automated algorithmic approach to identifying asynchrony incidence, where, as noted, treatment for different types can be very different.

More specifically, Fig. 12 provides an example of P1 with the continuous monitoring of asynchronous rate over breaths and time. The fa and rt asynchronies show 15% and 36% (51% in total) fraction of total breaths, respectively, at BC 300, whilst the non-asynchrony rate is only 33% with smaller grey area or range than the non-grey (red and blue) areas. However, the non-asynchrony grey area increased to 65% with the asynchronous red and blue areas dropping down to 31% at BC500 due to sedation administration. Compared to the commonly used asynchrony index defined as the number of asynchronous breaths divided by the total number of breaths [27], Fig. 12 thus offers quantified changes and qualified visualization of different types of asynchrony and non-asynchrony phenomena over breath and time for a long-term bedside monitoring metric.

Although the variability in the data includes recruitment manoeuver, different types of asynchronies, change of asynchrony for the same patient over time, different patient diagnosis, and different ventilator modes, the patient variability for broad cohort can be much more significant than the current data cohort. It should be noted patient condition changes regardless of the duration of time period, whilst the significance of changes depends on patient conditions and applied treatment. Therefore, the validation data of 30–40 min represent a comparable duration to clinical bedside visual inspection for MV adjustment, which was mainly used to validate the potential utility of the method in clinical practice.

Compared to popular machine learning methods for asynchrony detection of one or a few types of asynchrony due to its limitation of requiring a very high number of unbiased test breaths [39], this method was validated against a wider range of asynchrony covering all 7 types reported as the most common asynchrony types with similar or better accuracy. In addition, machine learning methods tend to only use similar pressure and/or flow waveform for training feature extraction, whilst this study offers a different approach and insight from using coupled hysteresis response as a potential training feature. The use of hysteresis loops and behaviour for training a predictive model has already proven its efficacy [57]. Therefore, the proposed method could be integrated with machine learning approaches if the data density was high enough for effective training, or used directly as presented, where Eqs. (5)–(11) define the key PV loop features necessary to discriminate asynchrony types.

A further advantage of the explicit model-based method over black-box methods is its ability to provide waveform reconstruction, thus enabling estimations of lung mechanics critical for clinical interpretation of MV settings [13, 31]. Reconstruction of unaffected PV loops has been demonstrated for reverse triggering [61], and the method is generalizable to those asynchrony types presented here. In all cases, information theory holds, and severely altering PV loops with asynchronous, patient-driven inputs can result in inadequate information for accurate reconstruction.

Human inspection is a common standard method for asynchrony examination, but requires more time than the identification algorithm presented, even for skilled doctors. Human input is thus not feasible for breath-to-breath, real-time continuous monitoring. However, combining intermittent clinician evaluation with breath-to-breath estimation from the identification algorithms presented could provide greater confidence in the resulting clinical decisions. Although the identification method can be implemented automatically and provide a detailed statistical analysis on asynchrony incidence and type, as shown in Figs. 1, 2, 3, 4, 5, 6, 7, 8, 9, 10 and 11, the resulting adjustment of MV settings will still likely be conducted by clinicians, in particular because the association between asynchrony type and its therapeutic response(s) remains unclear [35].

It is noted that the measurement of heterogeneous air distribution may provide a more detailed regional lung responses to ventilator for more accurate evaluation [44]. However, current ventilators can only provide global pressure and flow measurements, which may not reflect the regional or heterogeneous distribution of lung volume and condition variations. CT scans could measure heterogeneous distribution, whilst unrealistic for bedside monitoring. Electrical impedance tomography (EIT) is a promising and non-invasive tool for regional lung volume assessment, but its accuracy and maturity remain to be validated [15, 23], unable to provide the long-term consistent monitoring. Therefore, the global data measurement directly obtained from the ventilator is the most commonly available data for bedside evaluation at any time during the MV treatment, which is the focus of this study to characterize the pattern of the global hysteresis mechanics for asynchrony identification.

In terms of limitations, asynchrony patterns vary across MV modes and patient lung mechanics for the same asynchrony type. The patient numbers for validation is 11, which includes limited scenarios and MV modes. In addition, more patient data whilst undergoing RMs and associated increased paralysis and sedation could clearly show the difference between asynchrony and non-asynchrony breaths for each patient, enabling a more confident ground truth for validation.

Human inspection can be subjective with classification performance limited to the pre-defined patterns or thresholds of different asynchrony types, without considering their significance or relevance for clinical changes in MV care. A large retrospective observational study on MV mode selection for 559,734 cases demonstrated significant heterogeneity between individual ICU units, hospitals and over periods [25]. Thus, the classification made in this proof-of-concept study might be different from other units or doctors. For example, minor ineffective efforts identified as asynchrony in this trial may be considered as non-asynchrony in others due to clinically insignificant differences. The criterion difference thus yields different incidence rates in the identified statistical analysis, leading to variable changes of settings per clinical standard. Therefore, there is a need for future study in the field to consider more detailed classifications of asynchrony magnitude, in addition to incidence and type, relevant to specific clinical standard needs to be further studied to improve the clinical utility of this type of algorithm- and model-based monitoring.

Equally, it is worth noting visual inspection has been and is currently still one of the major clinical means for bedside asynchrony assessment. In particular, a jury of experienced, well-trained clinicians can provide accurate assessment of asynchrony via visual inspection given sufficient jury time [17], even whilst noting this level of time and experience is not expandable to regular monitoring by all clinical staff, so this method is not realistic for bedside monitoring and thus clinically problematic. Hence, the proposed method aims to automate the current clinical process in a real-time fashion with competitive efficacy to doctors to enable an equity monitoring of each of the MV patients in the same ICU per consistently, whilst its validation against EAdi is limited in this study.

## Conclusions

This study proposed a readily automated, algorithm-based asynchrony identification method based on a hysteretic lung mechanics model and hysteresis loop analysis method to detect the incidence and type of asynchrony for the 7 most common type of patient–ventilator asynchrony. Validation using 5500 breaths of data from 11 ICU patients over several different mechanical ventilation modes demonstrates very good accuracy compared to clinical visual inspection by three ICU clinicians. The results show the potential clinical advantage of breath-to-breath, real-time model-based monitoring of asynchrony by type, and provide the foundation for model-based reconstruction of unaltered PV loops to assess asynchrony magnitude. Overall, the identification method could assist particularly less-experienced clinicians to achieve more efficient and accurate bedside monitoring of asynchrony and MV care in general.

## Methods and materials

### Hysteresis loop analysis and piecewise regression models

**Fig. 13:**
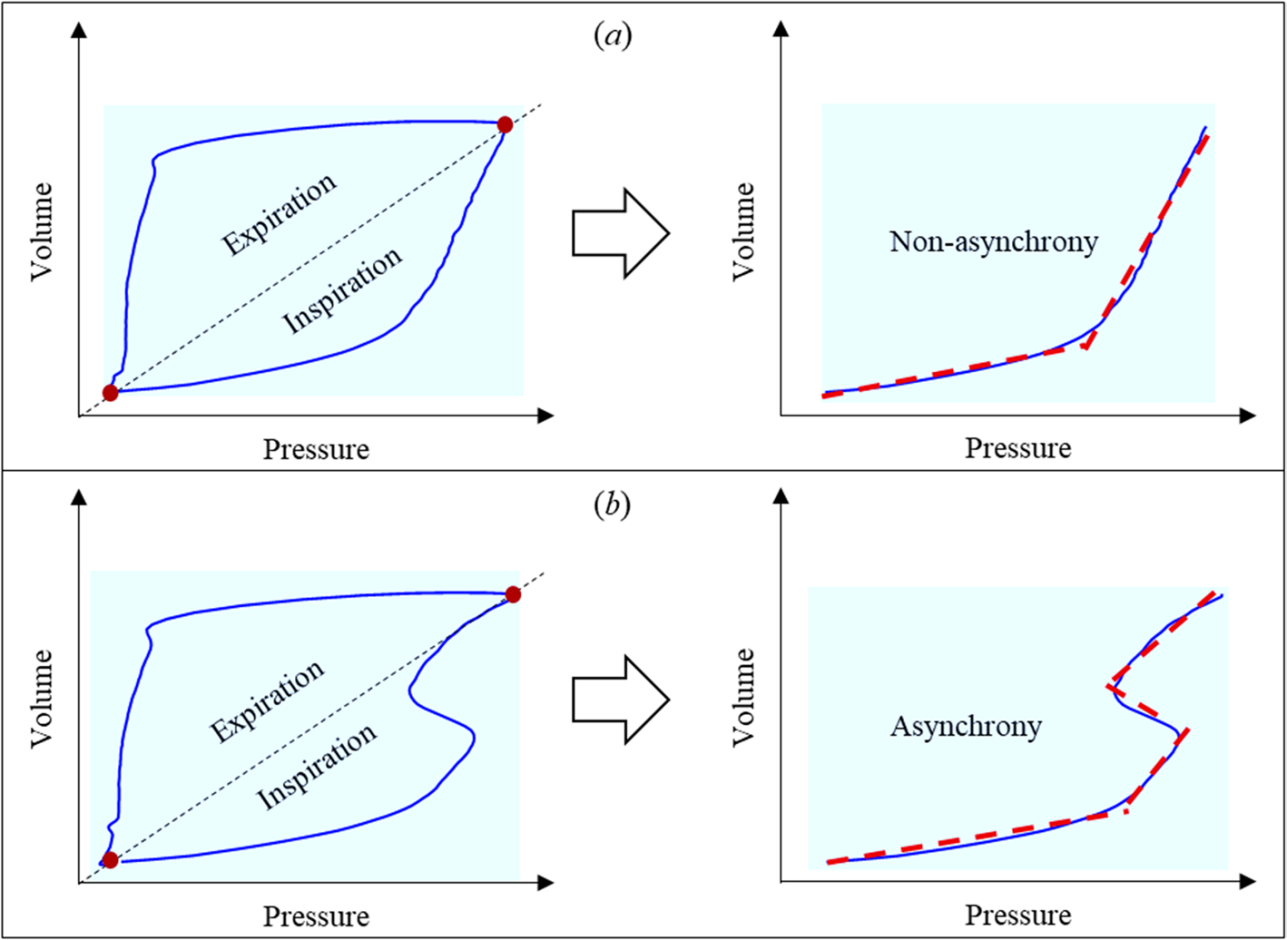
Example of segmentation of a PV loop breathing cycle during inspiration for: **a** no asynchrony, and **b** a case with asynchrony

Hysteresis observed in various dynamic systems plays a crucial role in system performance analysis, and captures nonlinearities and energy dissipation associated with changes of mechanics and response [2, 45, 56, 58, 59]. In particular, this hysteretic behaviour for mechanical ventilated ICU patients is captured by the PV loop, a readily measured set of signals. In general, the PV loop can be divided into inspiratory and expiratory half-cycles using the turning point at maximum volume. A piecewise regression model can then be used to approximate the nonlinear hysteresis behaviour of each half-cycle [62]. For example, the inspiratory half-cycle of a ventilated PV loop without asynchrony can be approximated by a two-segment model, as seen in Fig. 13a, whilst a PV loop with asynchrony requires more segments for an accurate approximation, as shown in Fig. 13b. Similar plots can be created for expiratory asynchrony, or asynchrony across both breathing cycle phases.

In particular, a *r*-segments piecewise regression model can be written:

1 $$ \begin{aligned} P(i) & = {k_{s1} V(i) + P_{b1} + e(i)} \quad {V({N_{0} }) \le V(i) \le V({N_{1} })} \\ &= {k_{s2} V(i) + P_{b2} + e(i)} \quad {V({N_{1} }) < V(i) \le V({N_{2} })} \\ & \quad \quad \quad \quad \quad \vdots \quad \quad\quad\quad\quad\quad \vdots \\ & = {k_{sr} V(i) + P_{br} + e(i)} \quad {V({N_{r - 1} }) < V(i) \le V({N_{r} })}\end{aligned} ,$$

where *P* and *V* are the measured MV pressure and volume;* k*_*s*1_, *k*_*s*2_, …, *k*_*sr*_ are the local elastance for divided breathing phase during inspiration and expiration; *P*_*b*1_, *P*_*b*2_, …, *P*_*br*_ are the interception in pressure axis of PV loop; *e* is random error between the measured airway pressure and the fitted pressure due to measurement noise and/or model uncertainty; *N*_0_ = 1 is the first point of the breath and *N*_*r*_ = *N* is the number of total observations for a single breath; *V*(*N*_1_), …, *V*(*N*_*r*−1_) are the volume breakpoints dividing the PV loop cycle into *r*-segments of breathing phase.

Importantly, the number of segments, *r*, breakpoints, and elastance values for each segment can correspondingly define a unique pattern. Thus, the identification of asynchrony type can be converted for the identification of the number of segments and their model parameter values. This approach holds whether asynchrony occurs during inspiration, expiration, or across both breathing cycle phases.

To determine the number of segments “*r*”, a hypothesis test based on *F*-distribution [3] is proposed between a null hypothesis of *r* segments and the alternative hypothesis of *r* + 1 segments, which requires the calculations of two *F*-ratios *F*(3|2) and *F*(4|3):

2 $$ F\left( {3|2} \right) = \frac{{{\text{RSSE}}\left( {V\left( {N_{0} } \right),V\left( {N_{1} } \right),V\left( {N_{2} } \right)} \right) - \mathop {\min }\limits_{1 \le i \le 2} \left\{ {\mathop {\inf }\limits_{{V\left( {N_{i - 1} } \right) \le \tau \le V\left( {N_{i} } \right)}} {\text{RSSE}}\left( {V\left( {N_{0} } \right), \ldots ,V\left( {N_{i - 1} } \right),\tau ,V\left( {N_{i} } \right), \ldots ,V\left( {N_{3} } \right)} \right)} \right\}}}{{\sigma^{2} }}, $$

3 $$ F\left( {4|3} \right) = \frac{{{\text{RSSE}}\left( {V\left( {N_{0} } \right),V\left( {N_{1} } \right),V\left( {N_{2} } \right),V\left( {N_{3} } \right)} \right) - \mathop {\min }\limits_{1 \le i \le 3} \left\{ {\mathop {\inf }\limits_{{V\left( {N_{i - 1} } \right) \le \tau \le V\left( {N_{i} } \right)}} {\text{RSSE}}\left( {V\left( {N_{0} } \right), \ldots ,V\left( {N_{i - 1} } \right),\tau ,V\left( {N_{i} } \right), \ldots ,V\left( {N_{4} } \right)} \right)} \right\}}}{{\sigma^{2} }}, $$

where RSSE is the residual sum of squared errors for regression analysis and *σ* the model variance estimated from the mean RSSE under the assumption of non-asynchrony. Note, normal inspiration and expiration half-cycles are assumed to comprise two segments divided by the lower inflection point and upper inflection point, respectively [43]. Thus, a non-asynchrony breath is also assumed to be a two-segment piecewise regression model in the work for each of these phases. in specific, *RSSE* for an *r*-segment regression is defined:

4 $$ {\text{RSSE}}\left( {V\left( {N_{0} } \right), \ldots ,V\left( {N_{i - 1} } \right), \ldots ,V\left( {N_{r} } \right)} \right) = \min \left\{ {\mathop \sum \limits_{{j_{1} = N_{0} }}^{{N_{1} }} \left( {P\left( {j_{1} } \right) - k_{s1} V\left( {j_{1} } \right) - P_{b1} } \right)^{2} + \cdots + \mathop \sum \limits_{{j_{i} = N_{i - 1} }}^{{N_{i} }} \left( {P\left( {j_{i} } \right) - k_{si} V\left( {j_{i} } \right) - P_{bi} } \right)^{2} + \ldots + \mathop \sum \limits_{{j_{r} = N_{r - 1} }}^{{N_{r} }} \left( {P\left( {j_{r} } \right) - k_{sr} V\left( {j_{r} } \right) - P_{br} } \right)^{2} } \right\}, $$

where *j*_1_, *j*_*i*_ and *j*_*r*_ are the data points in the 1st, *i*th and *r*th segment of the piecewise regression model, respectively. To find the minimum value of RSSE(*V*(*N*_0_), …,* V*(*N*_*i*−1_),…, *V*(*N*_*r*_)), a grid search method of testing all possible combinations of breakpoints *V*(*N*_1_), …, *V*(*N*_*r*−1_) [62] are implemented with associated model parameters *k*_*s*_ and *P*_*b*_ calculated via Eq. (4).

If the calculated *F* ratios in Eqs. (2) and (3) are smaller than the critical value, the null hypothesis test is then accepted. Otherwise, the alternative hypothesis test is accepted to consider using more segments to better approximate the half-cycle. Note the identification of the proposed piecewise linear model and model parameters are automatically implemented without requiring human input.

### Asynchrony patterns in lung hysteretic responses

**Fig. 14:**
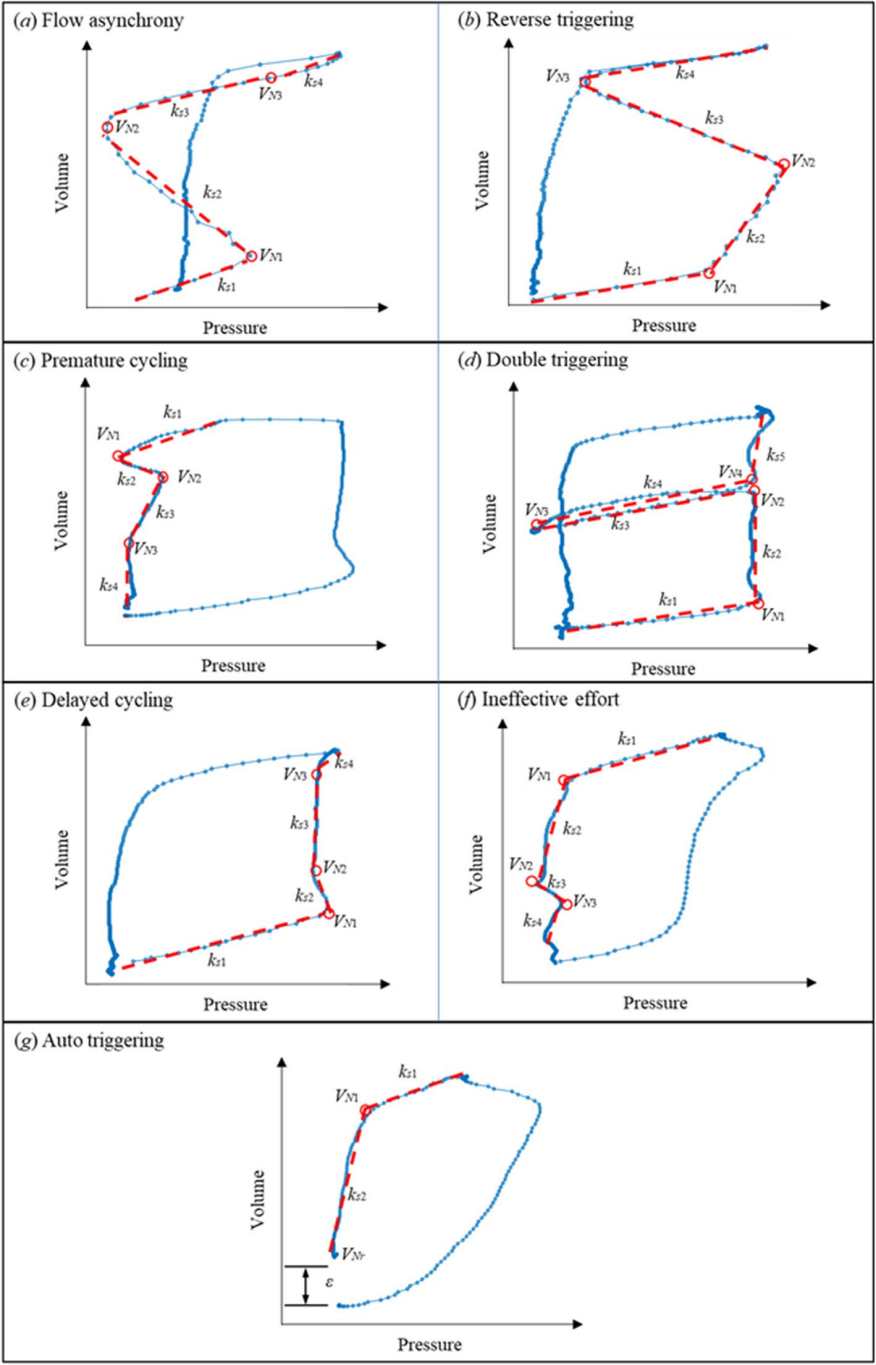
Hysteretic characteristic for asynchrony

Measured, patient-specific PV loops are approximated using the identified piecewise linear model with calculated breakpoints and regression parameters. Different numbers, *r*, of segments and parameter values/ranges determine a specific shape and model characteristics of the PV loop. Thus, the specific pattern created by different types of asynchronies can be explicitly interpreted by the change of hysteresis mechanics identified from PV loop analysis. Figure 14 shows the typical PV curves of the seven types of asynchronies using the real patient data collected from the clinical trial. In particular, identification of the seven most common types of asynchronies [14, 20, 21, 24, 38] based on the shape of the approximated PV loop and the calculated model parameters are implemented as follows:

#### Type 1: Flow asynchrony (fa)

Flow asynchrony occurs when patients’ flow demand exceeds the setting inspiration flow rate, typically creating a pressure drop in the beginning of inspiration without distorting the flow waveform. The appearance of flow asynchrony is shown in Fig. 14(a), corresponding to a 4-segment model defined:

5 $$ r = 4, k_{s1} > 0, k_{s2} < 0, k_{s3}  > 0, k_{s4} > 0. $$

#### Type 2: Reverse triggering (rt)

Reverse triggering is a type of asynchrony due to a reflexive neural response triggered by the passive ventilator insufflation. The occurrence of reverse triggering is delayed from ventilator-triggered (not patient-triggered) inspiration, and thus can lead patient effort to persist through the inspiration phase and into expiration, as shown in Fig. 14b. This type of asynchrony can be identified:

6 $$ r = 4, k_{s1} > 0, k_{s2} > 0, k_{s3} < 0, k_{s4} > 0. $$

#### Type 3: Premature cycling (pc)

For both patient triggering and ventilator triggering with late patient effort (reverse triggering), patient effort can be longer than ventilator set inspiration time, causing premature cycling. Due to continued patient inspiratory effort during expiration, premature cycling is normally observed in the beginning of the expiratory phase with a pressure depression and a jump of flow. The flow jump would thus create a slower decrease of volume. Therefore, the depressed pressure and the slow decline of volume cause a negative-slope segment *k*_*s*2_ in the early stage of expiration, as seen in Fig. 14c.

In addition, the breakpoint *V*_*N*1_ for the premature cycling segment is not left enough to create a second breath, which should be identified near the beginning of expiration. Therefore, using all these aspects, premature can be determined using

7 $$ r = 4, k_{s1} > 0, k_{s2} < 0, k_{s3} > 0, k_{s4} > 0, V_{N0} - V_{N1} < V_{T} /2, $$

where *V*_*T*_ is the tidal volume of the breath.

#### Type 4: Double triggering (dt)

Double triggering is defined by two consecutive ventilated breaths, wherein expiration of the first breath is not complete before the second. Similar to premature cycling, double triggering occurs when patient inspiration time is longer than the ventilator set inspiration time. However, the occurrence of double triggering requires much greater patient effort to overcome the set ventilation threshold, which leads to the extra 2–3 PV loop segments, as shown in Fig. 14d. Therefore, detection of double triggering can be converted into the identification of inspiration segments *r* > 4 regardless of the elastance values for each segment, yielding

8 $$ r = 5, k_{s1} > 0, k_{s1} > k_{s2} , k_{s3} > k_{s2} , k_{s4} > k_{s5} , P_{N3} < {\text{PEEP}}, P_{N2} > P_{N3} , P_{N4} > P_{N3} . $$

#### Type 5: Delayed cycling (dc)

In contrast to premature cycling and double triggering, if patient inspiratory time is shorter than ventilator set inspiration time, delayed cycling asynchrony appears with air trapping and a sudden rise of pressure at the end of inspiration. Thus, a sudden change of slope should be observed at the end of inspiration in the PV loop, leading to a separate segment with elastance value* k*_*s*4_ much higher than the previous segment *k*_*s*3_, as shown in Fig. 14e. Therefore, delayed cycling is defined:

9 $$ r = 4, k_{s1} > 0, k_{s2} < 0, k_{s3} > 0, k_{s4} > 0, k_{s4} > 2k_{s3} . $$

#### Type 6: Ineffective effort (ie)

Ineffective effort is one of the most common asynchrony types and can occur during both inspiration and expiration, although it is more common (78%) during expiration [52]. Ineffective effort results from patient effort not strong enough to trigger a ventilated breath, and thus normally appears as relatively small changes in pressure and flow. Similar to premature cycling, ineffective effort leads to a pressure drop and flow jump, whilst tending to be seen near the end of expiration, which is different from premature cycling. Thus, the negative-slope segment is expected to occur for *k*_*s*3_, as shown in Fig. 14f. Therefore, the detection of ineffective triggering is defined using

10 $$ r = 4, k_{s1} > 0, k_{s2} > 0, k_{s3} < 0, k_{s4} > 0, V_{N0} - V_{N2} > V_{T} /2. $$

#### Type 7: Auto triggering (at)

Auto triggering is a type of asynchrony causing an unexpected breath delivery, which does not match the set respiratory frequency and is not triggered by patient effort. It is normally an outcome of air leaking in the ventilator circuit or air trapping due to chronic obstructive pulmonary disease or obstructive ventilatory defect [1, 42]. No abrupt drop of pressure would be expected for auto triggering asynchrony. In addition, air leakage creates a leak of volume during expiration, leading to a gap between the baseline volume and the end expiratory volume, as shown in Fig. 14g. Thus, auto triggering is detected via the volume gap in PV loop, as defined using

11 $$ r = 2, k_{s1} > 0, k_{s2} > 0, V_{Nr} - V(0) > \varepsilon . $$

It should be noted Fig. 14 is not representative of all possible appearance forms of patient asynchrony in clinical practice. For example, the number of segments for flow asynchrony during inspiration was identified as 4 segments in Fig. 14a, whilst it could still be identified as flow asynchrony with 3 segments for cases with a clear pressure drop in the beginning of the inspiration. However, these 7 types of asynchronies cover the most typical asynchronies reported in other research [14, 20, 21, 24, 38].

### Clinical data

Clinical patient data were collected from eleven patients in ICU of the Fourth Hospital of Hebei Medical University in China. Ethics approval for data collection was granted by the local hospital ethics committee with number 2021KY131. Patients were ventilated using a Draeger Evita V300 ventilator. Pressure and flow data were recorded using respiratory mechanics monitoring tool CURESoft connected to the ventilator [51]. All data were recorded at a sampling rate of 100 Hz.

Asynchrony incidence and type were identified for 500 breaths (30–40min) for each patient. Per prior observational trials [5, 52], these breaths were inspected by three ICU doctors from the Key National Clinical Specialist in the Fourth Hospital of Hebei Medical University, China for each patient. In particular, the individual breath was first divided in CURESoft [51], and presented to the doctors with both pressure, flow and PV loop waveforms for asynchrony evaluation. The screening of both pressure and flow data can be adjusted over a variable period of time window per clinician request. Asynchronies were evaluated and voted independently by the jury of 3 experienced and trained doctors. The baseline label type for comparing with the proposed identification method was determined with more than two votes. Patient demographics are presented in Table 3 showing a wide range of ages, condition, and ventilation modes. Of the 11 adults, 4 are female, which is a typical ratio, with age ranging from 50 to 89 years old.

### Analyses

The identification algorithms for asynchrony incidence and types are implemented automatically via computer program software Matlab R2019b. Identification sensitivity and specificity were validated against the clinical inspection results from three ICU doctors, where sensitivity assesses the ability to accurate detect and classify asynchrony, whilst specificity assesses the ability to accurately assess normal breaths as having no asynchrony. Sensitivity and specificity are calculated:

12 $$ {\text{Sensitivity}} = \frac{{{\text{TP}}}}{{{\text{TP}} + {\text{FN}}}}, $$

13 $$ {\text{Specificity}} = \frac{{{\text{TN}}}}{{{\text{TN}} + {\text{FP}}}}, $$

where TP is the true positive defined as the match of agreement for the specified type between the algorithm identification and clinical inspection, whilst TN is the true negative defined as the match of disagreement for the specified type between the algorithm identification and clinical inspection. FN is the false negative indicating the algorithm failed to identify the breath as the asynchrony type specified by clinical inspection. FP is the false positive representing the algorithm identified the breath as the specified type, but it was classified as asynchronous by clinical inspection.

Therefore, overall accuracy can be defined using these elements from Eqs. (12)–(13) as the number of accurate assessments divided by the total:

14 $$ {\text{Accuracy}} = \frac{{{\text{TP}} + {\text{TN}}}}{{{\text{TP}} + {\text{FN}} + {\text{TN}} + {\text{FP}}}}. $$

Identification accuracy was evaluated for each patient separately to test robustness across different patient conditions and MV settings. It was also calculated over all patients to evaluate the overall performance of the identification method in this proof-of-concept cohort.

## Data Availability

The datasets generated and analyzed during the current study are not publicly available for privacy reasons but anonymized data are available from the corresponding author on reasonable request.
